# Renal Cell Carcinoma Metastases to the Pancreas and the Thyroid Gland: A Case Report and Review of the Literature

**DOI:** 10.7759/cureus.3667

**Published:** 2018-12-01

**Authors:** Marco Khiella, Michael A Maximus, Sameh A Fayek

**Affiliations:** 1 Miscellaneous, Loyola University, Chicago, USA; 2 Miscellaneous, Cairo University, Cairo, EGY; 3 Surgery, University of North Texas Health Science Center, Fort Worth, USA

**Keywords:** pancreatic metastases, thyroid metastases, renal cell carcinoma

## Abstract

Renal cell carcinoma (RCC) is known for having unpredictable clinical behavior. Metastases can occur in unusual locations with a long-time lag after the treatment of the primary cancer. Despite being a sign of poor prognosis, aggressive metastasectomy may prolong survival. Presented is a case of delayed sequential metastases of RCC to the pancreas and the thyroid gland that occurred eight years after the radical nephrectomy. Both were resected. A history of remote nephrectomy for RCC is important and may be suggestive of metastatic disease. Ultrasound-guided fine needle aspiration can be diagnostic and helps in decision-making. Aggressive surgical intervention when possible is recommended.

## Introduction

Renal cell cancer (RCC) is known for its unpredictable behavior. Spontaneous regression of metastases has been reported, and patients may survive for years with metastases [1]. RCC metastasize mainly to lung, bone, and liver; rare sites include skin, thyroid, adrenal gland, and pancreas. [2]. Tumors in these sites in patients with prior RCC should raise concern of metastatic disease. In select patients with good performance status, slow growing, indolent tumors presenting with localized disease, resection should be considered.

Here in we present a case with delayed sequential metastasis of renal cell cancer to unusual sites with successful surgical treatment.

## Case presentation

**Figure 1:**
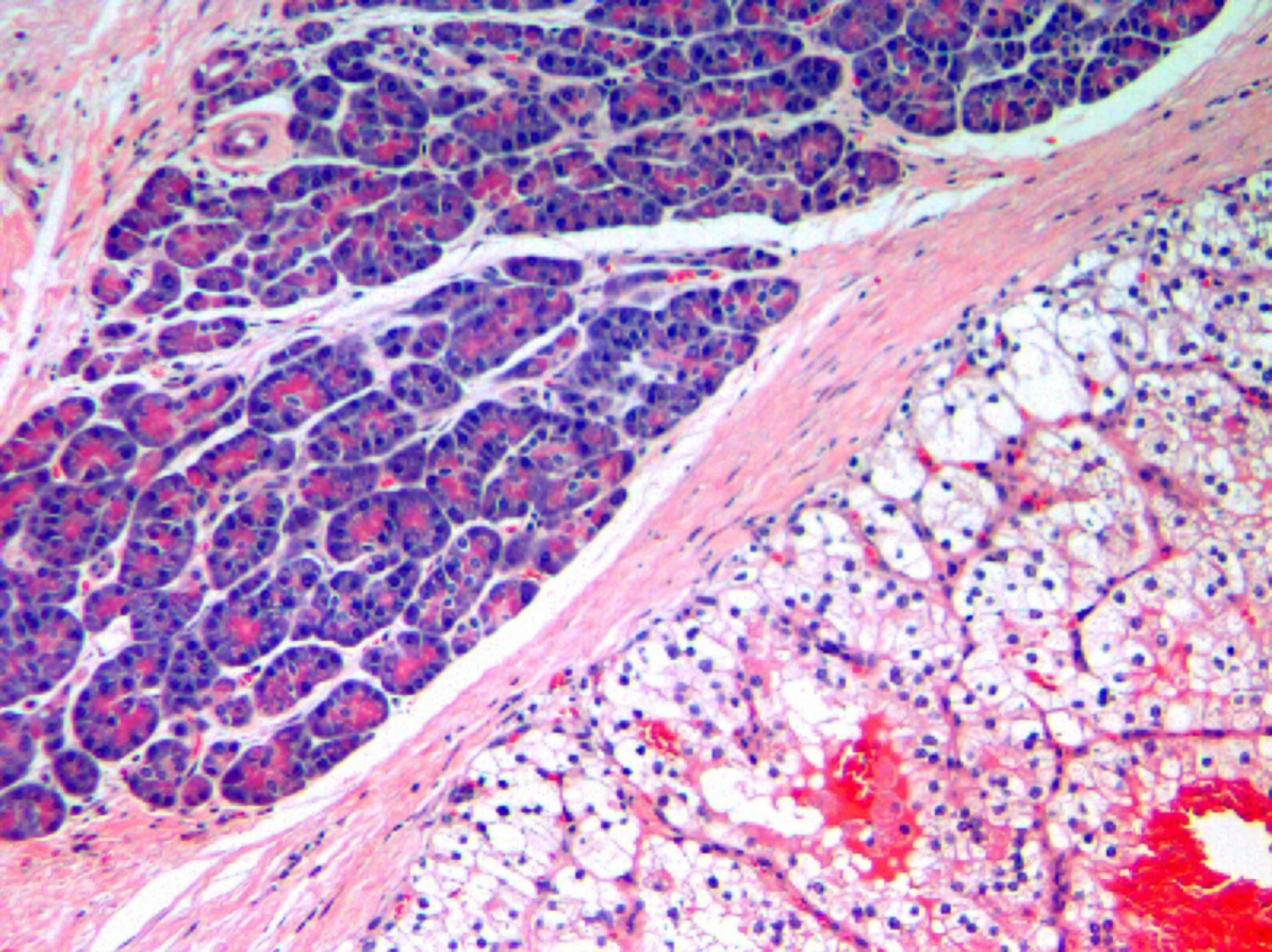
Metastatic renal cell cancer to the pancreas, Hematoxylin and eosin stain: pancreas acinar cells (blue - left upper side), renal cell cancer (vacuolated cells - right lower side)

**Figure 2:**
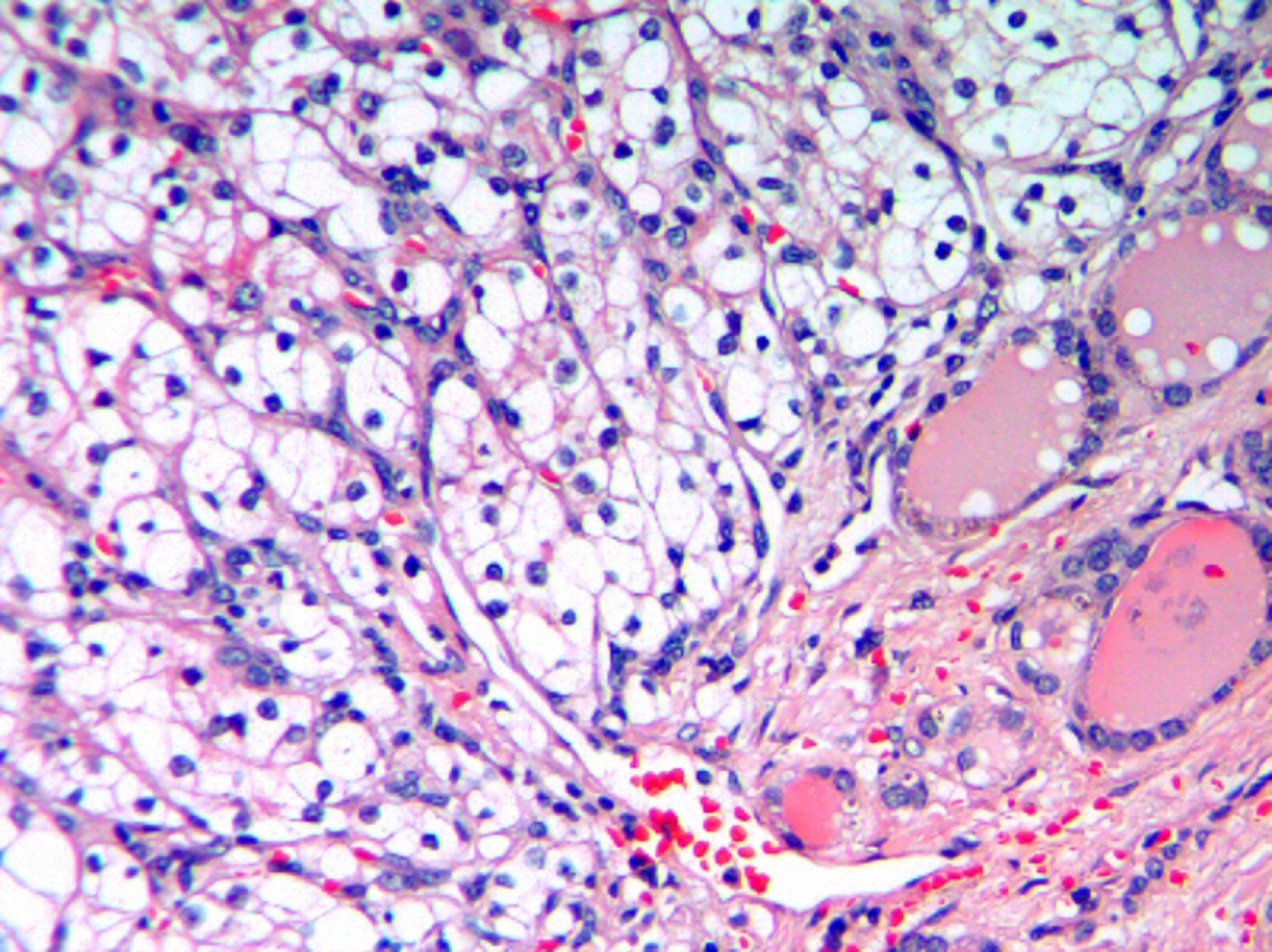
Metastatic renal cell cancer to the thyroid gland, Hematoxylin and eosin stain: renal cell cancer cells (vacuolated - left upper side), thyroid follicles with central colloid (right lower side)

A 73-year-old female presented with a new 3 cm mass in the pancreatic head that was found on an annual surveillance computerized tomography (CT) scan eight years after a radical left nephrectomy for renal cell carcinoma (RCC). Her initial nephrectomy was for an 8 cm clear cell RCC that had one positive periaortic lymph node (LN). The patient had no further treatment and denied abdominal pain but reported progressive weight loss (>60 lbs) over the last several months. She was not jaundiced and there was no evidence of duodenal or biliary duct obstruction on the CT scan. Other diagnostic modalities (endoscopic retrograde cholangiopancreatography (ERCP) and magnetic resonance imaging (MRI)) confirmed the pancreatic head mass. A bone scan and a chest X-ray were negative for metastatic disease. The patient underwent a diagnostic laparoscopy with pelvic washing and biopsies from peripancreatic tissue, celiac, splenic and periportal lymph nodes. There was no evidence of peritoneal, omental or hepatic spread and all biopsies were negative for malignancy. A pancreaticoduodenectomy (Whipple procedure) was performed for presumptive pancreatic cancer. Section through the specimen showed multiple solid yellowish necrotic and hemorrhagic areas (0.5 - 2.2 cm). The histologic exam was consistent with metastatic clear RCC (Figure 1), two of nine peripancreatic LN were positive for metastases. There was no neoplastic thrombus in the pancreatic duct and the margins were free from disease. Immunohistochemical stain showed tumor focally positive for cytokeratin 7 and keratin AE1/AE3 but negative for cytokeratin 20 and carcinoembryonic antigen. The tumor stained strongly positive with vimentin; all consistent with RCC. The pathology was identical to the slides from initial nephrectomy. Four months later, a gradually enlarging right lobe thyroid nodule was noticed associated with dysphagia. The nodule was cold on radionuclide scanning and solid on ultrasound, measuring 2.9 x 2.6 x 2 cm. Doppler showed marked increase in vascular flow. A CT scan of the neck, chest, and abdomen failed to demonstrate other lesions. Fine needle aspiration cytology (FNAC) was suggestive of metastatic RCC. A right hemithyroidectomy was performed (the left lobe was removed years ago for benign disease). Section through the tumor revealed a solid, homogenous, bulky yellowish nodular neoplasm. The histologic exam was consistent with metastatic RCC (Figure 2). The patient tolerated both procedures and has done well.

## Discussion

RCC is known for its unpredictable behavior. Spontaneous regression of metastases has been reported, and patients may survive for years with metastases [1]. RCC metastasize mainly to lung, bone, and liver; rare sites include skin, thyroid, adrenal gland, and pancreas [2, 3].

Metastases account for only 2%-3% of solid pancreatic tumors [2]. Although rare and poorly known [1,4], RCC is recognized to metastasize to the pancreas with slight male predominance [5]. Other pancreatic metastases are frequently small and found only by microscopic examination on autopsy. The slow growth of RCC explains the greater frequency with which its metastases are discovered in clinical setting [6]. RCC pancreatic metastasis could be solitary metastasis or could occur as part of widespread LN and visceral involvement [6]. Lesions can occur anywhere in the pancreas [5,6] and can be solitary or multiple [2]. Even a single pancreatic metastasis is usually a manifestation of systemic disease as its spread is hematogenous [6].

A long interval between nephrectomy and metastatic disease is common [1]. Pancreatic metastases may occur synchronously with RCC [4]. The average duration is 10 years [2] after the nephrectomy; however, this can range from few years [4,5] to as late as 27 years [2]. The diagnosis must be considered with a previous history of RCC [1]. In 50% of cases, metastases are discovered during radiologic surveillance [6] or radiographic exam for another condition [1]. Symptomatic patients may present with abdominal pain [3,4], jaundice, weight loss [2], upper-GI tract hemorrhage [4] or obstruction because of duodenal invasion [2]. Rarely patients present with diabetic ketoacidosis or malabsorption [2]. Usually physical exam is normal, occasionally an epigastric mass is palpable [2]. The prognosis of pancreatic RCC metastases is better than that of primary pancreatic adenocarcinoma or metastases of other origin, underscoring the importance of a preoperative diagnosis as good results may be obtained with surgery [2,7].

Pancreatic RCC metastases are hypervascular. This accounts for their radiologic appearance, which is like that of neuroendocrine tumors, whereas primary pancreatic adenocarcinoma is hypovascular. On ultrasound (US), the lesion appears hypoechogenic or exceptionally cystic. CT scan may be highly suggestive showing rounded lesions with well-defined contours that show intense, heterogeneous enhancement with hypodense areas after intravenous contrast injection. CT may show also tumor extension into the pancreatic duct [8]. Endoscopic ultrasound (EUS) is the most sensitive study especially for small isodense lesions that could be missed on CT and MRI [1,2]. EUS appearance is characteristic and differs from that of primary adenocarcinoma, with posterior or peripheral enhancement indicating a hypervascular structure [2]. However, occasionally the mass mimics a malignant primary tumor or cystic tumor [2]. Although EUS-guided needle biopsy is considered the technique of choice for cytologic and /or histopathologic sampling of solid pancreatic tumors, its role in pancreatic metastases is not clearly defined [2]. Due to the hypervascularity, biopsies are difficult to interpret, being highly hemorrhagic with no tumor found or mimicking a high-grade vascular neoplasm [8]. The continuous negative pressure of aspiration seems to destroy the typical clusters of cells with their abundant vacuolated cytoplasm and round nuclei with prominent nucleoli. Sampling technique modifications, namely short aspiration with low negative vacuum pressure, might improve preoperative diagnosis [2].

Aggressive surgical treatment for solitary metastatic lesions is advocated [6]. Just as RCC may extend into the renal vein, the pancreatic metastases can grow into the pancreatic duct seeking the path of least resistance. [8]. Vascular and LN involvement may occur with variable incidence [2, 6]. Resection with adequate margins is usually possible and may result in 30% survival rate [2, 4]. In experienced centers this is associated with low morbidity and mortality and can offer some patients their only chance of long-term survival [5]. Adjuvant chemo / endocrine therapy should be considered [6].

The thyroid gland has a rich blood supply [3]. In patients who died of malignancy, thyroid metastases are not uncommon at autopsy with rates from 1.2% to 24% [9], but these are rare in clinical practice and few require surgery [4]. The most common sites of primary tumors are the breast (21%), kidneys (12%), and lung (11%) [4]. Most cases (64%-85%) of metastatic RCC to the thyroid have evidence of a prior primary tumor [4,7], so history of prior malignancy is important in evaluating patients with a thyroid nodule [3, 5, 9]. The time from nephrectomy ranges from 3-19 years [4, 10] with an average of 8-9 years [7]. Rarely thyroid metastases are the initial presentation [6,7]. Commonly, a solitary nodule is found (83%) that is cold on scintigraphy [3]. Symptoms of tracheal compression [3] or dysphagia [4] may be present. On US a well-demarcated hypoechoic mass containing high-echo spots representing small calcifications is typically seen. CT may reveal a low-density mass containing small calcifications [9]. FNAC with immunohistochemical staining may strongly suggest a clear cell carcinoma metastasis to the thyroid [9].

Although metastasis to the thyroid gland often indicates poor prognosis, aggressive treatment may be effective, especially for RCC. [10] Total thyroidectomy, with or without radiation, is the procedure of choice especially in the presence of obstructive symptoms. [3]. This may result in prolonged survival; however, the mean survival is around 34 months [3]. Most patients (64%) will die with disseminated malignancies [6, 7]. With lung secondaries, addition of chemotherapy may result in a good clinical response with arrest of progression [3]. With dissemination, palliative and endoscopic intervention (e.g. placement of an endotracheal stent) are justified to improve quality of life and preserve the airway [11].

Histologically, the tumor is composed of polygonal cells with clear cytoplasm, distinct cell membranes, and small compact eccentric nuclei within a rich vascular network [7]. Thyroid gland clear cell tumors are uncommon, and even though metastatic RCC to the thyroid is rare, it must be considered in cases of a thyroid gland with clear cell neoplasm [7]. Using immunohistochemistry, RCC metastasis can now be identified unequivocally from a primary follicular thyroid carcinoma by a combination of TTF-1, thyroglobulin, and CD 10. [12] Thyroglobulin immunohistochemistry is always negative in the foci on metastatic RCC [4, 6, 7, 9]. Diastase-sensitive, periodic acid-Schiff-positive material in (61%) and /or Oil Red O-positive material (14%) were noted in metastatic RCC [7].

## Conclusions

This report presents a case of RCC with long disease-free interval and unusual metastatic sites. A history of nephrectomy for RCC, even dating back many years, is important in evaluating patients with pancreatic and thyroid gland tumors. In this reported case, the pancreatic metastasis was followed by the thyroid gland secondaries. Surgical excision is the treatment of choice when feasible.
